# A complement receptor C5a antagonist regulates epithelial to mesenchymal transition and crystallin expression after lens cataract surgery in mice

**Published:** 2011-04-19

**Authors:** Rinako Suetsugu-Maki, Nobuyasu Maki, Timothy P. Fox, Kenta Nakamura, Richard Cowper.Solari, Craig R. Tomlinson, Hongchang Qu, John D. Lambris, Panagiotis A. Tsonis

**Affiliations:** 1 Department of Biology and Center for Tissue Regeneration and Engineering at Dayton, University of Dayton, Dayton, OH; 2 Departments of Medicine and Pharmacology & Toxicology, Dartmouth Hitchcock Medical Center, Norris Cotton Cancer Center, Dartmouth College, Lebanon, NH; 3 Department of Pathology and Laboratory Medicine, University of Pennsylvania Medical School, Philadelphia, PA

## Abstract

**Purpose:**

To evaluate the effects of complement employing a mouse model for secondary cataract.

**Methods:**

The role of complement receptor C5a (CD88) was evaluated after cataract surgery in mice. An antagonist specific to C5a receptor was administered intraperitoneally to mice. Epithelial to mesenchymal transition (EMT) was evaluated by alpha-smooth muscle actin (α-SMA) staining and proliferation by bromodeoxyuridine (5-bromo-2'-deoxyuridine, BrdU) incorporation. Gene expression patterns was examined by microarray analysis and quantitative polymerase chain reaction (QPCR).

**Results:**

We found that administration of a C5aR antagonist in C57BL/6J mice decreases EMT, as evidenced by α-SMA expression, and cell proliferation. Gene expression by microarray analysis reveals discreet steps of gene regulation in the two major stages that of EMT and lens fiber differentiation in vivo. A hallmark of the microarray analysis is that the antagonist seems to be a novel stage-specific regulator of crystallin genes. At week two, which is marked by lens fiber differentiation genes encoding 12 crystallins and 3 lens-specific structural proteins were severely down-regulated.

**Conclusions:**

These results suggest a possible therapeutic role of an antagonist to C5aR in preventing secondary cataracts after surgery. Also these results suggest that crystallin gene expression can be regulated by pro-inflammatory events in the eye.

## Introduction

A major complication of cataract surgery is the formation of secondary cataracts [1]. After lens fiber removal, some lens epithelial cells do remain attached in the capsular bag that is left behind to hold in place the artificial lens. In many cases these lens epithelial cells proliferate and transdifferentiate to mesenchymal cells, thus clouding the artificial lens1. This complication requires costly laser treatment. In the past few years it was found that mice and rats are in fact good models for such studies [2-4]. After performing cataract surgery in mice (and rats) it was found that a substantial part of the lens is regenerated. However, early in this process epithelial to mesenchymal transition (EMT) does take place as well. Consequently, this system can be used to study EMT because of the vast genetic resources existing for these animals. In our previous studies we found, by microarray analysis, that several members of the complement system are upregulated during the early steps of lens regeneration, which is also characterized by EMT [5]. Also, complement activation has been linked with EMT of renal proximal tubular epithelial cells leading to renal fibrosis [6]. We have reasoned that inhibiting the function of such molecules might impede EMT. As a first step we have decided to analyze the effect of complement component 5 (C5) in this system.

Our results clearly show that an antagonist of C5a receptor delays proliferation and EMT significantly both in vivo and in vitro. Examination of global gene expression is consistent with the effects of the antagonist on the cellular events taking place during lens regeneration. In particular it is shown that this antagonist might be a novel stage-specific regulator of crystallin synthesis.

## Methods

### Cataract surgery and C5aR antagonist administration

C57BL/6J mice (eight weeks old, female purchased from Jackson laboratory, Bar Harbor, ME) were anesthetized with either intraperitoneal or subcutaneous injection of Ketamine (95 mg/kg; Sigma-Aldrich, St. Louis, MO) and Xylazine (14.3 mg/kg; Sigma-Aldrich). Mice were also subcutaneously given the analgesic Buprenorphine (1 mg/kg; Sigma-Aldrich) preemptively. After corneal incision anterior capsulerectomy was performed. The lens core and fiber cells were broken by forceps and removed from the lens capsule gently. The capsule was washed with 1× PBS containing Mg^2+^ and Ca^2+^ to remove fiber cells. A specific antagonist of C5a receptor (PMX53), cyclic hexapeptide Ac-Phe-[Orn-Pro-dCha-Trp-Arg], was used [7]. Following surgery, mice were injected with the peptide (1 mg/kg bodyweight, in PBS) into the abdominal cavity every 2 days. Control mice were injected with Ac-Phe-[Orn-Pro-dCha-Ala-dArg] at the same concentration, also intraperitoneally. Control and experimental peptides were not injected in the eyes. Samples were taken 1, 2, or 3 weeks post-surgery and examined histologically or processed for microarray analysis. The authors confirm adherence to the ARVO statement for the use of animals in ophthalmic & vision research.

### Evaluation of EMT and cell proliferation

Eyeballs were collected and fixed in 4% Paraformaldehyde (Acros Organics, Morris Plains, NJ) overnight, at 4 °C, and processed for paraffin embedding. Sections of 15 μm were stained with hematoxylin and eosin (HE) or processed for immunohistochemical staining with mouse alpha-smooth muscle actin Ab (alpha-SMA Ab; 1/500 dilution; Sigma-Aldrich), O/N at 4 °C. This step was followed by addition of secondary FITC or Cy3 conjugated anti-mouse IgG (1/100 dilution) for 90 min at room temperature. 5-Bromo-2’-deoxyuridine (BrdU; #B5002; SIGMA) was administered to mice 1 day before collecting eyes by i.p. injection, at 500 mg/kg bodyweight. Sections were treated with 3N HCl for 10 min at room temperature before blocking and 1st antibody application (mouse anti-BrdU, #MAB3510, 1/100 dilution; Millipore, Billerica, MA). Pictures of immunohistochemistry were taken using a microscope (BX51; Olympus, Tokyo, Japan) with a CCD camera (Cool SNAP cf2; Photometrics, Tucson, AZ) and imaging software (Metamorph; Molecular Devices, Eugene, OR). For statistical analysis we used the Student’s *t*-test.

### Microarray analysis

Microarray hybridization and analysis were performed as described in Medvedovic et al. [5]. Eyes were collected 1, 2, and 3 weeks after surgery and stored in RNAlater (Ambion, Austin, TX) at −70 °C. Mice were injected with C5aR antagonist or control peptide every 2 days i.p. Experimental (C5) and control mRNAs were labeled with Cy3 or Cy5 fluorescent dyes using the Agilent Low RNA Input Fluorescent Linear Amplification Kit (Agilent, Santa Clara, CA). Two-color samples were prepared and hybridized using Agilent’s Multi-Pack Gene Expression Microarray platform according to Agilent instructions and using Agilent reagents. The design consisted of 60-mer oligonucleotide probes arrayed in four 44 K individual microarrays printed on a single glass slide, each covering the whole genome of the mouse (catalog number G4122F, design ID 014868; Ambion, Austin, TX). Each microarray is composed of approximately 41,000 unique biologic features and several hundred positive and negative controls. Each biologic sample was run in quadruplicate with a dye-flip design, scanned with an Agilent Microarray Scanner System and further processed with the Agilent Feature Extraction Software.

The Limma Bioconductor package was used to identify differentially expressed genes. Data preprocessing was performed by subtracting background from signal intensities and separately normalizing each of the arrays with the loess method [8-10]. Differential expression was assessed by means of the linear models and empirical Bayes method proposed by Smith et al. [11]. The KEGG spider web-based tool was used to identify enriched Gene Ontology (GO) categories in the lists of differentially expressed genes [12]. The R language and environment for statistical computing [13] was used to manipulate and graph data.

### In vitro culture of lens capsular bags

Culture of adult mouse lens capsular bags was performed as described previously with some modifications [14]. Lenses were removed from adult mice eye by dissection. They were then treated with 0.25% Tripsin/EDTA for 5min at room temperature and rinsed with culture medium, 10% FBS/DMEM including Penicillin/Streptomycin. Then an incision was made in the anterior lens capsule, from which the lens fibers cell mass was removed by forceps. The capsular bags were pinned on a 3 cm cell culture dish (#430165; Corning Lowell, MA) with 6 to 8 entomological pins (D1; Watkins and Doncaster, Kent, UK) and incubated with medium for 30 min at 37 °C. Following that capsules were photographed using a microscope (TS100; Nikon, Tokyo, Japan) with a CCD camera (Cool SNAP cf2; Photometrics, Tucson, AZ) and imaging software (Metamorph; Molecular Devices, Eugene, OR). This was our day 0 samples. We then treated the cultured capsular bags with either the C5aR antagonist or control peptide (final 1 μg/ml) and incubated them at 37 °C in a 5% CO_2_ environment. We followed the cultures and recorded progression of cell proliferation migration and EMT at 1, 2, and 3days after initiation of the treatment. To examine proliferation BrdU (final 50 μg /ml) was added for 3 h before fixation.

### Quantitative PCR

Regenerated lenses were collected using a mouth pipette. RNA was purified using Nucleospin (Macherey-Nagel, Bethlehem, PA). Room temperature (RT) reaction was performed with a first-strand cDNA synthesis kit (Amersham Bioscience, Piscataway, NJ) using an oligo(dT) primer. qPCR was performed using a iQ SYBR green supermix (Bio-Rad, Hercules, CA) and the following primers: To quantitate the expression of each gene, Ct values were compared to a standard curve generated using a series of dilutions of cloned cDNAs. Specific PCR amplifications were confirmed by melting curve analysis and by sequencing. crystallin alpha A (*CryaA*) F: 5′-GAG ATT CAC GGC AAA CAC AAC-3′, R: 5′-CAT TGG AAG GCA GAC GGT AG-3′; crystallin alpha B (*CryaB*) F: 5′-ACT CAA AGT CAA GGT TCT GGG-3′, R: 5′-GGG ATG AAG TGA TGG TGA GAG-3′; crystallin beta B2 (*CrybB2*) F: 5′-CCA TTC CCA CGA GCT CAG-3′, R: 5′-TCG CCC TTT TCA AAC ACA AAC-3′; glyceraldehyde-3-phosphate dehydrogenase (*GAPDH*) F: 5′-GCC TCG TCT CAT AGA CAA GAT G-3′, R: 5′-CAG TAG ACT CCA CGA CAT AC-3′.

## Results and Discussion

**Figure 1:**
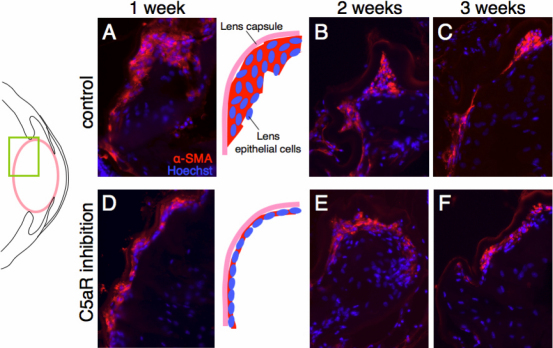
Effects of C5aR antagonist on EMT of lens epithelial cells cataract surgery in vivo. EMT was assessed by staining of sections with alpha-SMA 1, 2, and 3 weeks after removal of lens fibers. Sections were counter-stained with Hoechst. All sections are compared from the same region (posterior part of the eye as shown by the green rectangle in the illustration). Note the decrease in alpha-SMA signal one week after the operation in the C5aR antagonist-treated animals when compared with the control (**A**, **D**; also depicted in the adjacent illustration). Alpha-SMA positive area was gradually decreased even in control animals 2 and 3weeks post-operation (**B**, **C**, **E**, and **F**) showing similar levels in both groups. 20×.

**Figure 2:**
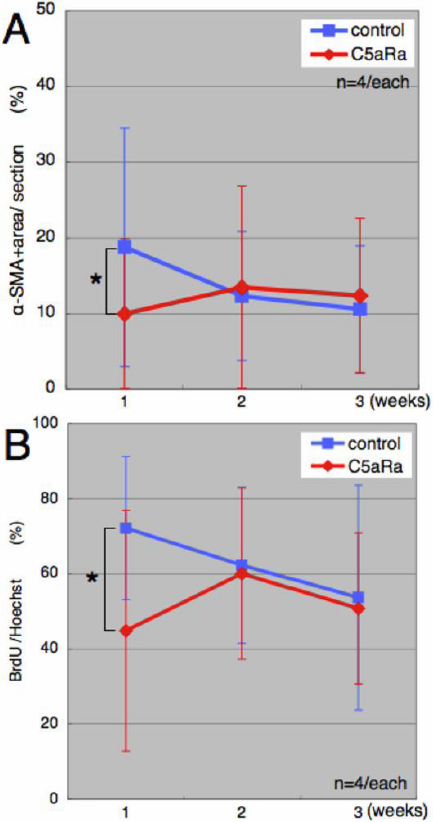
Quantitation of EMT and cell proliferation. Quantitation of a-SMA positive area (**A**) and BrdU-positive cells (**B**) in control (blue) and C5aR antagonist-treated animals (red). These graphs were made from sections received in the experiment described in Figure 1. Panel **A** confirms that C5aR antagonist caused delay of EMT but also affected proliferation of lens epithelial cells as well. The asterisks indicate the time when the results are statistically significant, p=0.042<0.05 for α-SMA and p=0.02<0.05 for BrdU/Hoechst.

After removal of the lens fibers, regeneration of the lens was followed for three weeks. As it has been reported before during this period of time epithelial cells transit to mesenchymal cells and also differentiate to lens fiber cells as well [4]. In fact it was found that by week 1 after the operation there was quite extensive EMT, which was later subsided as lens fibers were differentiated. In other words we have two critical stages, one during the first week marked by upregulation of extracellular matrix and EMT and the other (week 2 and 3) marked by upregulation of lens structural proteins and lens differentiation. We examined samples at week 1, 2, and 3 after the operation. Samples were stained with alpha-SMA to detect EMT in both controls and C5aR antagonist-treated animals. The results are shown in Figure 1. Immunohistochemistry showed a marked decrease of EMT in C5aR antagonist treated mice. Alpha-SMA positive cells were less and remain as a monolayer in the treated animals, while in the untreated ones the positive cells are multilayered. To better quantitate the results of Figure 1 we measured the area of alpha-SMA stained regions in the sections. We were able to show that at week 1 time point there was a clear and statistically significant difference in the number of EMT positive cells, obviously affected by the treatment with the C5aR antagonist (Figure 2A). Likewise we observed same kind of difference in the proliferating cells as indicated by the incorporation of BrdU. It is clear from these results that C5aR is important for the early stages of the regeneration process, which is dominated by EMT and that its inhibition brings the levels of EMT to levels observed at later stages, 2 and 3 weeks post-operation.

**Figure 3:**
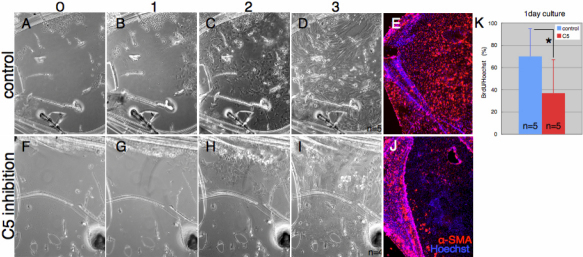
Effects of C5aR antagonist on cell migration, proliferation and EMT in lens capsular bag cultures. **A**-**C**: Cell migration of lens epithelial cells treated with control peptide viewed at day 0, 1, 2, and 3, respectively. **E**: a-SMA staining in control peptide-treated capsular bags 3 days in culture. **F**-**J**: Similar series with C5aR antagonist-treated capsular bags. Note that in capsular bags cultured with C5aR antagonist, migration of cells was slower than control. Also note that EMT as was evidenced by a-SMA staining was significantly reduced in the treated cultures (compare **E** to **J**). **K**: BrdU positive cells in both control experimental cultures (statistically significant by *t*-test; p<0.05).

To better examine this effect of C5aR antagonist we performed an in vitro experiment where capsular bags were cultured. It has been show before that isolation and culture of lens capsular bags is a great model to study migration, proliferation and EMT [1]. Capsular bags were cultured for three days in the presence or absence of the C5aR antagonist. Migration was observed and we found that it was inhibited in the C5aR antagonist-treated cultures. This can be easily seen in Figure 3A-D (control) and Figure 3F-I (C5 antagonist-treated). EMT was also significantly inhibited as can be seen in Figure 3E (control) and Figure 3J (experimental). Note that proliferation was also significantly affected in the treated capsular bags (Figure 3K). It should be noted here that after day 1 (and especially in control cultures) cells form multi-layers obscuring correct evaluation of proliferation (thus we present the effects on proliferation only at day 1.

So far our results show conclusively that C5 antagonist treatment leads to reduced proliferation and EMT of the lens epithelial cells attached to the capsule. Our purpose for this was to assess possible therapeutic value of the treatment.

**Figure 4:**
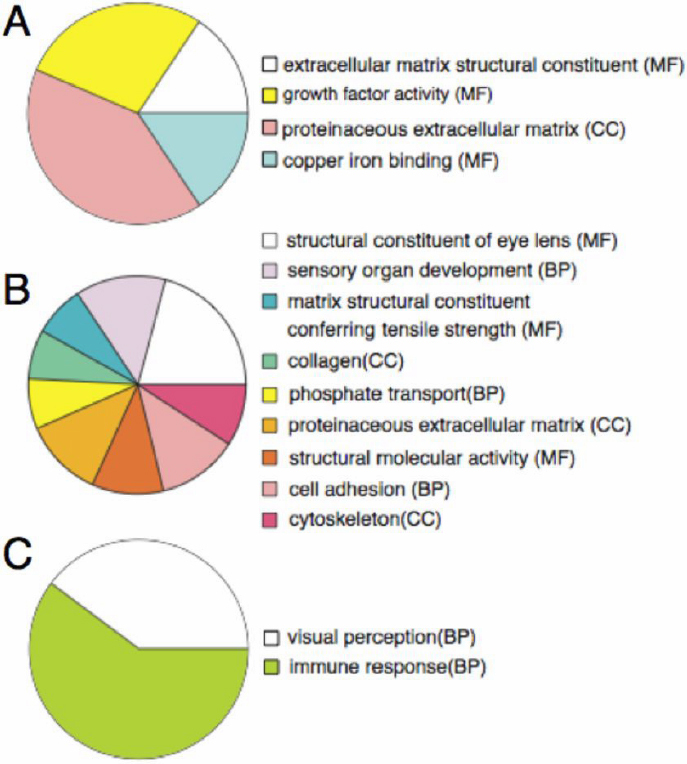
Microarray analysis. The complete lists are presented in Appendix 1. For each gene the degree of down- or upregulation due to the treatment is recorded. In this Figure the pies show differentially regulated genes grouped according to their function. Upper pie: regulated genes at week 1 after operation. Note that most regulated genes due to the C5aR antagonist treatment are related to extracellular matrix most likely indicating active migration. Middle pie: regulated genes at week 2 after operation. Note that the largest group is the one containing structural constituents of eye lens, indicating regulation of crystallin genes (see also Table 1). Lower pie: regulated genes at week 3 after operation. Only genes related to visual perception and immune response are regulated (as being statistically enriched). CC, BP, and MF are the three domains of Gene Ontology, Cellular Component, Biologic Process, and Molecular Function, respectively.

**Figure 5:**
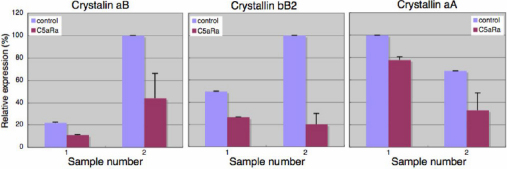
QPCR analysis of three crystallin genes. Two different samples were used from operated animals two weeks after surgery. For each sample the analysis was in triplicates. Note that expression of these crystallin genes was down regulated in all cases due to C5 antagonist treatment, as was the case in the microarray analysis. Statistics are as follows: *CryaB*-1: p=0.0000114343<0.05, *CryaB*-2: p=0.0000296783<0.05, *CrybB2*-1: p=0.0000598278<0.05, *CrybB2*-2: p=0.0002057<0.05, *CryaA*-1: p=0.005616049<0.05, *CryaA*-2: p=0.000385042<0.05.

In a previous study [5] we had analyzed in details gene expression patterns in control mice undergoing lens regeneration after cataract surgery and compared them with the intact lens. As in the present study, regenerating lens had been isolated, RNA was extracted and gene expression patterns were compared at week 1, 2, and 3. It was striking from that analysis that week 1 lens was dominated by upregulation in extracellular factors as well as complement components (that is why we also selected to study the complement system in more details in the present study). Also at week 1 and 2 there was a significant down-regulation of crystallins. However, after that period and especially at week-3 genes that encode for the structural constituents of the lens fibers were significantly upregulated to the levels of intact lens. That study confirmed that during the lens regeneration event we had an early stage with domination of EMT and later stages of lens fiber differentiation and lens regeneration. An interesting paper using the same mouse model for cataract surgery has shown that the operation induces pro-inflammatory gene expression in retina. Among the regulated genes are members of the complement system [15]. It is obvious then that the effects of the operation could affect gene expression in other tissues of the eye. Likewise, the effects of the C5aR antagonist could also be mediated by inflammation-related factors. C5 has been implicated in many different cellular events by controlling proliferation, as during liver regeneration [16,17]. We were therefore interested to examine gene expression in the control and C5aR antagonist-treated animals. After operation and treatment we collected eyes at week 1, 2, and 3, isolated RNA and used it as probes for microarray analysis. The microarray experiment was planned to compare the control with the C5aR antagonist-treated mice at the different time points (control week 1 versus C5aR antagonist week 1, and so on). This experiment does not compare week 1 expression with week 2 or week 3.We then listed the genes that were differentially regulated due to the treatment. The results were quite interesting and consistent with the events that we have observed so far. The results are shown in Figure 4 and the different groups in the pies are the groups that are regulated because the C5aR-antagonist treatment. A hallmark in the regulated genes was down regulation of crystallin genes. At week 1 there was down-regulation of γD-crystallin and βB1-crystallin genes only. However, week 2 was marked by widespread down-regulation of several crystallin genes (Table 1). Apart crystallin genes, there was consistent regulation of genes depending on the events that take place. At week 1 and week 2 we observed that among the down regulated genes there were ones involved in cytoskeleton, fibrogenesis, myogenesis, and extracellular matrix while genes involved in inflammation, such as ones in interferon regulation and interleukin 1 beta were among the upregulated ones (Table 2, Table 3, Table 4, Table 5, and Appendix 1). Interestingly, there were only very few upregulated genes in the treated eyes at week 2. Finally by week 3 most of the regulation occurred in components of visual perception and immune response. Interestingly among the upregulated ones were aquaporin 5, important for lens transparency as well as cytoskeletal protein-encoding genes (Table 6, Table 7, and Appendix 1). In Table 2, Table 3, Table 4, Table 5, Table 6, and Table 7 only the top 25 regulated genes are shown and some genes of interest are highlighted in red (full list in Appendix 1). In Figure 4 the regulated gene groups are presented as parts of a pie. It is evident from the expression studies that gene regulation due to C5aR inhibition does follow a stage-specific pattern. First there is regulation of factors that are involved in extracellular matrix (important for cell migration) and proliferation and then there is regulation of lens fiber-specific genes. Our previous studies on gene expression during lens regeneration [5] had indicated that crystallin expression levels at week 3 after surgery were very similar to un-operated intact lens. So obviously, in our system, the most critical stage for lens fiber differentiation is week 2. And this is the time that we see most down-regulation of crystallin genes due to C5aR inhibition. To verify these results we also performed QPCR analysis using primers specific for *CryaB*, *CrybB2*, and *CryaA*. The results clearly showed that all these three genes were indeed down-regulated by the C5aR antagonist treatment (Figure 5). This interesting finding suggests a novel role of C5aR antagonist in stage-specific crystallin gene regulation. Collectively, our data indicate a novel role of C5aR antagonist in secondary cataract formation by negatively affecting both EMT and lens fiber differentiation of the remaining lens epithelia cells in the capsule and imply that regulation of the complement components might be therapeutically beneficial after cataract surgery. The mechanism, however, is elusive. Normally, the results should be attributed to C5aR-mediated signaling, but we were unable to confirm our results in experiments where we used capsular bags from C5aR^−/−^ mice, nor we observed expression of C5aR in the lens capsules (even though C5 was present; unpublished information). One explanation could be that this peptide does bind to other receptors, such as Mas-related G-protein coupled receptor member X2 (MrgX2) or orphan receptor MrgX3 [18]. Alternatively, at least in vivo the results could be explained by PMX53 mediated signaling in other eye tissues and action to the lens by effector genes or by mediation of inflammation-related factors. Future studies toward that direction will elucidate these issues.

**Table 1:** Regulation of crystallin genes due to C5 antagonist.

**Gene symbol**	**p value**	**C5**	**Control**	**C5/Control**	**Description**
**Week 1**					
Crygd	0.0295	0.857	4.8861	0.18	Mus musculus crystallin, gammaD
Crybb1	0.049	2.0434	3.8395	0.53	Mus musculus crystallin, beta B1
**Week 2**					
*Crygd*	0.0004	35.2252	77.7724	0.45	Mus musculus crystallin, gammaD
*Cryga*	0.0003	29.4933	63.9658	0.46	Mus musculus crystallin, gammaA
*Crygc*	0.0007	79.0894	165.1198	0.48	Mus musculus crystallin, gammaC
*Crybb3*	0.0064	36.1689	61.9493	0.58	Mus musculus crystallin, beta B3
*Crybb1*	0.0067	15.2235	25.577	0.60	Mus musculus crystallin, beta B1
*Cryaa*	0.0096	98.3355	161.5709	0.61	Mus musculus crystallin, alpha A
*Crybb2*	0.0099	121.6081	197.3289	0.62	Mus musculus crystallin, beta B2
*Cryba4*	0.013	85.449	136.8441	0.62	Mus musculus crystallin, beta A4
*Crygf*	0.0476	1.0844	1.7216	0.63	Mus musculus crystallin, gamma F
*Crygn*	0.028	2.6886	4.1197	0.65	Mus musculus crystallin, gamma N
*Cryab*	0.0361	182.0757	272.6681	0.67	Mus musculus crystallin, alpha B
*Cryba1*	0.0435	278.2914	407.5949	0.68	Mus musculus crystallin, beta A1
*Bfsp1*	0.0013	7.6191	14.4174	0.53	*Mus musculus* beaded filament structural protein in lens
*Lenep*	0.0179	0.8412	1.3748	0.61	*Mus musculus* lens epithelial protein
*Bfsp2*	0.0417	8.2683	12.1496	0.68	*Mus musculus* beaded filament structural protein 2 in lens

**Table 2:** Genes down-regulated 1week after operation.

**C5_1W**	**Control_1W**	**C5/Control**	**Description**
0.86	4.89	0.18	*Mus musculus* crystallin, gamma D (Crygd), mRNA [NM_007776]
1	3.78	0.27	*Mus musculus* sarcolipin (Sln), mRNA [NM_025540] Ca^2+^ transport
1.37	3.56	0.39	PREDICTED: *Mus musculus* myosin, heavy polypeptide 3, skeletal muscle, embryonic (Myh3), mRNA [XM_354614]
32.92	70.44	0.47	*Mus musculus* hemoglobin, beta adult major chain (Hbb-b1), mRNA [NM_008220]
19.02	40.27	0.47	Mouse alpha-globin mRNA. [M10466]
0.7	1.37	0.51	PREDICTED: *Mus musculus* sodium channel, voltage-gated, type II, alpha 1 (Scn2a1), mRNA [XM_909341]
2.04	3.84	0.53	*Mus musculus* crystallin, beta B1 (Crybb1), mRNA [NM_023695]
0.4	0.73	0.55	*Mus musculus* Rho GTPase activating protein 5 (Arhgap5), mRNA [NM_009706]
0.51	0.91	0.56	*Mus musculus* ring finger and SPRY domain containing 1 (Rspry1), mRNA [NM_026274]
65.76	114.52	0.57	*Mus musculus* procollagen, type I, alpha 1 (Col1a1), mRNA [NM_007742]
0.67	1.15	0.58	*Mus musculus* prostate stem cell antigen (Psca), mRNA [NM_028216]
0.6	1.03	0.58	*Mus musculus* matrilin 4 (Matn4), mRNA [NM_013592] ECM organization
0.53	0.91	0.58	*Mus musculus* round spermatid basic protein 1-like (Rsbn1l), mRNA [NM_001080977]
0.58	0.99	0.59	*Mus musculus* AT motif binding factor 1 (Atbf1), mRNA [NM_007496]
1.6	2.67	0.6	*Mus musculus* WAP four-disulfide core domain 3 (Wfdc3), mRNA [NM_027961]
0.82	1.29	0.63	*Mus musculus* cDNA sequence BC003885 (BC003885), mRNA [NM_198609]
0.77	1.2	0.64	*Mus musculus* solute carrier family 17 (anion/sugar transporter), member 5 (Slc17a5), mRNA [NM_172773]
0.53	0.81	0.65	*Mus musculus* RIKEN cDNA 1110061N23 gene (1110061N23Rik), mRNA [NM_176834]
0.41	0.63	0.65	*Mus musculus* runt related transcription factor 2 (Runx2), mRNA [NM_009820] Trnascription factor, mesenchymal stem cells
43.09	66.14	0.65	*Mus musculus* cytochrome P450, family 2, subfamily a, polypeptide 5 (Cyp2a5), mRNA [NM_007812]
2.14	3.28	0.65	*Mus musculus* myosin, heavy polypeptide 7, cardiac muscle, beta (Myh7), mRNA [NM_080728]
0.62	0.95	0.66	Riken cDNA C130021I20 gene [Source:MarkerSymbol;Acc:MGI:3639863] [ENSMUST00000065087]
1.19	1.8	0.66	*Mus musculus* osteomodulin (Omd), mRNA [NM_012050]
12.03	18.17	0.66	*Mus musculus* cytochrome P450, family 2, subfamily a, polypeptide 4 (Cyp2a4), mRNA [NM_009997]
0.51	0.77	0.66	RIKEN cDNA 4933437N03 gene [Source:MarkerSymbol;Acc:MGI:1914033] [ENSMUST00000035891]

**Table 3:** Genes upregulated 1 week after operation.

**C5_1W**	**Control_1W**	**C5/Control**	**Description**
1.34	0.47	2.83	*Mus musculus* arachidonate 15-lipoxygenase (Alox15), mRNA [NM_009660]
1.01	0.36	2.81	*Mus musculus* melanoma-derived leucine zipper, extra-nuclear factor (Mlze), mRNA [NM_031378]
0.95	0.43	2.23	*Mus musculus* cell death-inducing DFFA-like effector c (Cidec), mRNA [NM_178373]
5.69	2.87	1.98	*Mus musculus* Z-DNA binding protein 1 (Zbp1), mRNA [NM_021394]
1.25	0.66	1.91	*Mus musculus* 13 days embryo male testis cDNA, RIKEN full-length enriched library, clone:6030459M14 product:unclassifiable, full insert sequence. [AK031607]
1.18	0.64	1.83	*Mus musculus* coiled-coil domain containing 100 (Ccdc100), mRNA [NM_178686]
0.73	0.41	1.81	*Mus musculus* membrane-spanning 4-domains, subfamily A, member 4B (Ms4a4b), mRNA [NM_021718]
1.09	0.61	1.79	*Mus musculus* ras responsive element binding protein 1 (Rreb1), transcript variant 1, mRNA [NM_001013392]
0.74	0.43	1.72	*Mus musculus* interferon regulatory factor 1 (Irf1), mRNA [NM_008390]
0.62	0.36	1.7	*Mus musculus* CD5 antigen (Cd5), mRNA [NM_007650]
0.62	0.37	1.68	*Mus musculus* RIKEN cDNA 4930511J11 gene (4930511J11Rik), mRNA [NM_029070]
0.67	0.41	1.65	nucleotide binding protein 1 [Source:MarkerSymbol;Acc:MGI:1347073] [ENSMUST00000023146]
4.26	2.61	1.63	*Mus musculus* RIKEN cDNA 2310016F22 gene (2310016F22Rik), mRNA [NM_173743]
0.6	0.37	1.6	*Mus musculus* ubiquitin D (Ubd), mRNA [NM_023137]
3.44	2.16	1.59	*Mus musculus* myelin basic protein (Mbp), transcript variant 7, mRNA [NM_010777]
0.58	0.37	1.57	*Mus musculus* activity-dependent neuroprotective protein (Adnp), mRNA [NM_009628]
1.17	0.74	1.57	*Mus musculus* brain derived neurotrophic factor (Bdnf), transcript variant 1, mRNA [NM_007540]
0.61	0.39	1.56	*Mus musculus* proprotein convertase subtilisin/kexin type 1 (Pcsk1), mRNA [NM_013628]
1.83	1.18	1.55	*Mus musculus* cDNA sequence BC048679 (BC048679), mRNA [NM_183143]
0.61	0.4	1.54	*Mus musculus* translocase of outer mitochondrial membrane 40 homolog (yeast) (Tomm40), mRNA [NM_016871]
12.91	8.39	1.54	*Mus musculus* interferon inducible GTPase 1 (Iigp1), mRNA [NM_021792]
1.07	0.7	1.53	*Mus musculus* thyroid hormone receptor interactor 4 (Trip4), mRNA [NM_019797]
0.57	0.38	1.52	*Mus musculus* 0 day neonate thymus cDNA, RIKEN full-length enriched library, clone:A430106H13 product:tripartite motif-containing 35, full insert sequence. [AK020775]
0.58	0.38	1.52	*Mus musculus* transmembrane protein 48 (Tmem48), mRNA [NM_028355]
2.31	1.52	1.52	*Mus musculus* coagulation factor X (F10), mRNA [NM_007972]

**Table 4:** Genes down-regulated 2 weeks after operation.

**C5_2W**	**Control_2W**	**C5/Control**	**Description**
35.23	77.77	0.45	*Mus musculus* crystallin, gamma D (Crygd), mRNA [NM_007776]
2.61	5.72	0.46	*Mus musculus* complement factor D (adipsin) (Cfd), mRNA [NM_013459]
29.49	63.97	0.46	*Mus musculus* crystallin, gamma A (Cryga), mRNA [NM_007774]
79.09	165.12	0.48	*Mus musculus* crystallin, gamma C (Crygc), transcript variant 1, mRNA [NM_007775]
0.64	1.32	0.49	Unknown
7.62	14.42	0.53	*Mus musculus* beaded filament structural protein in lens-CP94 (Bfsp1), mRNA [NM_009751]
0.69	1.26	0.55	*Mus musculus* fibronectin 1 (Fn1), mRNA [NM_010233]
26.91	48.97	0.55	*Mus musculus* procollagen, type I, alpha 1 (Col1a1), mRNA [NM_007742]
4.43	8	0.55	*Mus musculus* lactase-like (Lctl), mRNA [NM_145835]
2.3	3.98	0.58	*Mus musculus* serine (or cysteine) peptidase inhibitor, clade F, member 1 (Serpinf1), mRNA [NM_011340]
36.17	61.95	0.58	*Mus musculus* crystallin, beta B3 (Crybb3), mRNA [NM_021352]
3.19	5.4	0.59	*Mus musculus* procollagen, type V, alpha 1 (Col5a1), mRNA [NM_015734]
2.83	4.79	0.59	*Mus musculus* fibromodulin (Fmod), mRNA [NM_021355]
15.22	25.58	0.6	*Mus musculus* crystallin, beta B1 (Crybb1), mRNA [NM_023695]
0.82	1.37	0.6	*Mus musculus* RAD21 homolog (S. pombe) (Rad21), mRNA [NM_009009]
0.73	1.2	0.6	*Mus musculus* X-linked lymphocyte-regulated 3B (Xlr3b), mRNA [NM_011727]
1.73	2.86	0.61	*Mus musculus* procollagen, type XII, alpha 1 (Col12a1), mRNA [NM_007730]
98.34	161.57	0.61	*Mus musculus* crystallin, alpha A (Cryaa), mRNA [NM_013501]
0.66	1.09	0.61	*Mus musculus* deltex 4 homolog (Drosophila) (Dtx4), mRNA [NM_172442]
5.79	9.5	0.61	*Mus musculus* fatty acid binding protein 4, adipocyte (Fabp4), mRNA [NM_024406]
0.84	1.37	0.61	*Mus musculus* lens epithelial protein (Lenep), mRNA [NM_020517]
1.39	2.27	0.61	*Mus musculus* 16 days embryo lung cDNA, RIKEN full-length enriched library, clone:8430418K19 product:gap junction membrane channel protein alpha 3, full insert sequence [AK136383]
121.61	197.33	0.62	*Mus musculus* crystallin, beta B2 (Crybb2), mRNA [NM_007773]
2.17	3.51	0.62	PREDICTED: *Mus musculus* gene model 96, (NCBI) (Gm96), mRNA [XM_129042]
0.56	0.9	0.62	*Mus musculus* syntaxin binding protein 5 (tomosyn) (Stxbp5), mRNA [NM_001081344]

**Table 5:** Genes upregulated 2 weeks after operation.

**C5_2W**	**Control_2W**	**C5/Control**	**Description**
0.89	0.45	1.97	Unknown
1.55	0.83	1.88	*Mus musculus* chitinase 3-like 3 (Chi3l3), mRNA [NM_009892]
1	0.64	1.56	*Mus musculus* interleukin 1 beta (Il1b), mRNA [NM_008361]

**Table 6:** Genes down-regulated 3 weeks after operation.

**C5_3W**	**Control_3W**	**C5/Control**	**Description**
18.14	55.19	0.33	*Mus musculus* crystallin, beta B1 (Crybb1), mRNA [NM_023695]
0.45	1.34	0.34	Unknown
0.84	2.22	0.38	*Mus musculus* myelin basic protein (Mbp), transcript variant 7, mRNA [NM_010777]
0.44	0.99	0.44	*Mus musculus* cell death-inducing DFFA-like effector c (Cidec), mRNA [NM_178373]
0.44	0.92	0.48	*Mus musculus* adiponectin, C1Q and collagen domain containing (Adipoq), mRNA [NM_009605]
0.77	1.49	0.52	*Mus musculus* adult retina cDNA, RIKEN full-length enriched library, clone:A930026N13 product:retinitis pigmentosa 1 homolog (human), full insert sequence. [AK044612]
0.89	1.62	0.55	*Mus musculus* LSM14 homolog B (SCD6, S. cerevisiae), mRNA (cDNA clone IMAGE:4458586), partial cds. [BC040823]
6.51	11.62	0.56	*Mus musculus* elongation of very long chain fatty acids (FEN1/Elo2, SUR4/Elo3, yeast)-like 4 (Elovl4), mRNA [NM_148941]
0.67	1.2	0.56	*Mus musculus* selenophosphate synthetase 2 (Sephs2), mRNA [NM_009266]
4.39	7.72	0.57	*Mus musculus* 0 day neonate eyeball cDNA, RIKEN full-length enriched library, clone:E130019F23 product:unclassifiable, full insert sequence [AK053456]
13.07	22.14	0.59	*Mus musculus* guanine nucleotide binding protein, beta 1 (Gnb1), mRNA [NM_008142]
0.55	0.92	0.59	*Mus musculus* adult retina cDNA, RIKEN full-length enriched library, clone:A930017E01 product:unclassifiable, full insert sequence. [AK044500]
0.83	1.4	0.59	*Mus musculus* pleckstrin homology domain containing, family F (with FYVE domain) member 2 (Plekhf2), mRNA [NM_175175]
1.56	2.64	0.59	*Mus musculus* gap junction membrane channel protein alpha 9 (Gja9), mRNA [NM_010290]
0.61	1.02	0.6	*Mus musculus* immunoglobulin superfamily, member 11 (Igsf11), mRNA [NM_170599]
1.51	2.5	0.6	*Mus musculus* zinc finger, DHHC domain containing 2 (Zdhhc2), mRNA [NM_178395]
0.63	1.04	0.61	*Mus musculus* RIKEN cDNA 9130404D08 gene (9130404D08Rik), mRNA [NM_028993]
0.48	0.78	0.61	*Mus musculus* RAR-related orphan receptor beta (Rorb), transcript variant 2, mRNA [NM_146095]
0.55	0.89	0.62	*Mus musculus* 12 days embryo spinal ganglion cDNA, RIKEN full-length enriched library, clone:D130077C01 product:unclassifiable, full insert sequence [AK141969]
0.67	1.08	0.62	*Mus musculus* shroom family member 2 (Shroom2), mRNA [NM_172441]
4.58	7.32	0.62	*Mus musculus* cone-rod homeobox containing gene (Crx), mRNA [NM_007770]
0.47	0.75	0.63	*Mus musculus* protein kinase, cAMP dependent regulatory, type II alpha (Prkar2a), mRNA [NM_008924]
0.77	1.23	0.63	*Mus musculus* 13 days embryo male testis cDNA, RIKEN full-length enriched library, clone:6030496O18 product:unclassifiable, full insert sequence [AK031720]
0.51	0.81	0.63	PREDICTED: *Mus musculus* golgi autoantigen, golgin subfamily b, macrogolgin 1, transcript variant 1 (Golgb1), mRNA [XM_148244]
0.68	1.07	0.63	*Mus musculus* 13 days embryo lung cDNA, RIKEN full-length enriched library, clone:D430019O20 product:unclassifiable, full insert sequence [AK084964]

**Table 7:** Genes upregulated 3 weeks after operation.

**C5_3W**	**Control_3W**	**C5/Control**	**Description**
47.14	24.78	1.9	*Mus musculus* keratin 16 (Krt16), mRNA [NM_008470]
59.19	33.03	1.79	*Mus musculus* expressed sequence AI661453 (AI661453), mRNA [NM_145489]
1.15	0.65	1.76	*Mus musculus* synaptosomal-associated protein 23 (Snap23), mRNA [NM_009222]
4.34	2.47	1.76	*Mus musculus* interferon inducible GTPase 1 (Iigp1), mRNA [NM_021792]
28.41	16.24	1.75	*Mus musculus* phosphatidylethanolamine binding protein 2 (Pbp2), mRNA [NM_029595]
1.71	0.98	1.75	*Mus musculus* Z-DNA binding protein 1 (Zbp1), mRNA [NM_021394]
3.28	1.89	1.73	*Mus musculus* S100 calcium binding protein A9 (calgranulin B) (S100a9), mRNA [NM_009114]
183.41	110.51	1.66	*Mus musculus* aquaporin 5 (Aqp5), mRNA [NM_009701]
3.6	2.18	1.65	*Mus musculus* actin, alpha 2, smooth muscle, aorta (Acta2), mRNA [NM_007392]
1.79	1.09	1.64	*Mus musculus* dihydrolipoamide dehydrogenase (Dld), mRNA [NM_007861]
1.02	0.62	1.64	*Mus musculus* ring finger protein 146 (Rnf146), mRNA [NM_026518]
61.8	37.62	1.64	*Mus musculus* Kruppel-like factor 4 (gut) (Klf4), mRNA [NM_010637]
3.07	1.88	1.63	*Mus musculus* RIKEN cDNA 1810019J16 gene (1810019J16Rik), transcript variant 2, mRNA [NM_133707]
61.67	38.14	1.62	*Mus musculus* RIKEN cDNA 1600029D21 gene (1600029D21Rik), mRNA [NM_029639]
4.59	2.83	1.62	*Mus musculus* purinergic receptor P2Y, G-protein coupled 2 (P2ry2), mRNA [NM_008773]
6.36	3.92	1.62	*Mus musculus* cadherin EGF LAG seven-pass G-type receptor 1 (Celsr1), mRNA [NM_009886]
2.1	1.3	1.62	*Mus musculus* macrophage stimulating 1 receptor (c-met-related tyrosine kinase) (Mst1r), mRNA [NM_009074]
2.15	1.34	1.61	*Mus musculus* potassium inwardly-rectifying channel, subfamily K, member 6 (Kcnk6), mRNA [NM_001033525]
46.63	28.89	1.61	*Mus musculus* myosin, heavy polypeptide 14 (Myh14), mRNA [NM_028021]
10.92	6.84	1.6	*Mus musculus* wingless related MMTV integration site 10a (Wnt10a), mRNA [NM_009518]
1.32	0.83	1.6	*Mus musculus* basic leucine zipper transcription factor, ATF-like 2 (Batf2), mRNA [NM_028967]
48.92	31.05	1.58	*Mus musculus* procollagen, type XVII, alpha 1 (Col17a1), mRNA [NM_007732]
134.36	85.11	1.58	*Mus musculus* envoplakin (Evpl), mRNA [NM_025276]
1.7	1.07	1.58	*Mus musculus* RIKEN cDNA 4931406C07 gene (4931406C07Rik), mRNA [NM_133732]
10.31	6.58	1.57	*Mus musculus* poly (ADP-ribose) polymerase family, member 14 (Parp14), mRNA [NM_001039530]

## Appendix 1. Data on the microarray analysis.

To access the data, click or select the words “Appendix 1.” This will initiate the download of a Microsoft Exel file (.xlsx).
